# Anthropological significance of *Tilia* trees in Japan

**DOI:** 10.1002/ece3.10303

**Published:** 2023-07-12

**Authors:** Daniel Janowski

**Affiliations:** ^1^ Department of Natural Environmental Studies The University of Tokyo Kashiwa Japan

**Keywords:** ecosystem services, *Tilia japonica*, *Tilia kiusiana*, *Tilia maximowicziana*, tree conservation

## Abstract

*Tilia* (Malvaceae) is a genus of deciduous trees widespread in the northern hemisphere. *Tilia* species native to Japan include three endemic species, *T. japonica*, *T. maximowicziana*, and *T. kiusiana*, as well as the more widespread *T. mandshurica*. Other species were also introduced, the most important being *T. miqueliana*, brought to Japan with the arrival of Buddhism and planted on temple grounds as Bodaiju. Both historically and at present, *Tilia* trees are valuable to the people in Japan. Some *Tilia* trees are considered sacred in both Buddhism and Shinto. They are also prominent ornamental and park trees, albeit less popular in Japan than in Europe. Japanese *Tilia* spp. are used in the manufacturing of honey, cosmetics, lumber (especially plywood and veneers), and traditional bast cloth. Many *Tilia* trees are significant hubs in pollinator and mycorrhizal networks, but research on Japanese *Tilia* ecology is scarce. Despite their importance, Japanese *Tilia* trees have received less scientific attention in comparison with European *Tilia* species. The most striking example is *T. kiusiana*, with virtually no scientific literature regarding the species (save for a series of publications studying its secondary metabolites and potential medical uses). Furthermore, most published resources concerning *Tilia* in Japan are available only in Japanese, restricting their accessibility. This review seeks to translate, collect, and organize the information available on Japanese *Tilia* species. By doing so, areas are highlighted where new studies are necessary. A better understanding of these important trees would also be instrumental in their conservation.

## Graphical abstract

Both historically and at present, *Tilia* trees are valuable to the people in Japan. However, despite their importance, Japanese *Tilia* trees received limited scientific attention compared with European *Tilia* species. This review highlights the various uses of *Tilia* trees and identifies the understudied aspects of their applications.

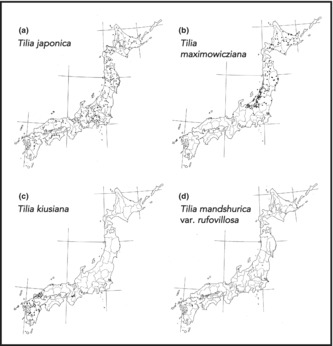

## 1 INTRODUCTION

The *Tilia* L. genus belongs to the Malvaceae family. It comprises around 23 known extant species of deciduous trees widely distributed in the Northern Hemisphere (Pigott, 2012). They are commonly known as basswood in North America, linden or lime in Europe, duànshù (椴树) in China, and shinanoki (シナノキ; 科の木) or bodaiju (ボダイジュ; 菩提樹) in Japan. *Tilia* trees are medium‐sized, growing up to 40 m in height, with a broad and rounded crown. Their leaves are alternate, simple, and usually heart‐shaped. Depending on the species, flower color ranges from white to green to yellow. They produce a strong, characteristic smell, are rich in nectar, and are insect‐pollinated. The *Tilia* fruit is round, woody nutlets attached to an elongated bract resembling a small leaf. *Tilia* can be either di‐ or tetraploid, a feature important in taxonomic identification (Pigott, 2012).

People have long valued *Tilia* species for numerous reasons. They are important honey plants, and humans have been already consuming *Tilia* honey during the Bronze Age (Kvavadze et al., 2006). Unifloral *Tilia* honey is known for its unique fragrance, flavor, and medicinal properties (Juan‐Borrás et al., 2014; Rogóż et al., 2022). In addition to honey, pharmacologically active compounds can be found in the flowers and leaves of many *Tilia* species. These compounds have a wide range of effects, including anti‐inflammatory, anti‐cancerogenic, and antidiabetic (Frezza et al., 2019; Kosakowska et al., 2015; Shimada et al., 2014). Recent studies suggest that *Tilia* secondary metabolites might also alleviate symptoms of depression (Martínez‐Hernández et al., 2021; Turrini et al., 2020).

*Tilia* wood is lightweight, soft, and easily workable. This makes it suitable for woodcarving and manufacturing musical instruments (De Jaegere et al., 2016; Pigott, 2012; Rowe & Blazich, 2008). Another *Tilia*‐derived material that has been used throughout history is bast—fibers obtained from tree bark (Böhlmann, 1971). In regions across North America, Europe, and Asia, *Tilia* bast was used to make ropes, textiles, and clothes (Oeggl, 2008).

In addition to their practical uses, *Tilia* are valued as city trees. Besides their aesthetic appeal, they are also surprisingly resistant to pollution and drought. As such, *Tilia* trees readily grow in urban environments (Pigott, 2012). While common in European urban areas (Andrianjara et al., 2021; Rahman et al., 2017; Wolff et al., 2019), *Tilia* trees are less often seen in Asian cities.

**FIGURE 1:**
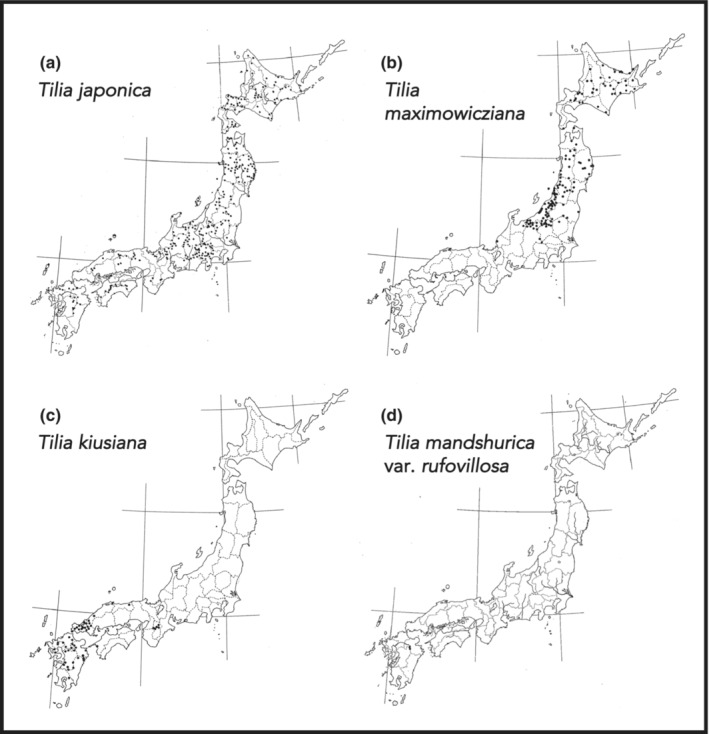
Distribution of native *Tilia* trees in Japan: (a) *Tilia japonica*; (b) *Tilia maximowicziana*; (c) *Tilia kiusiana*; (d) *Tilia mandshurica* var. *rufovillosa*. Black points represent recorded stands of the respective species. Distribution maps adapted from Kurata and Hamaya (1968–1976).

Three endemic species of *Tilia* are native to the Japanese islands. *Tilia japonica* (Miq.) Simonk. (シナノキ or アカシナ) is the most widespread and can be found in all of Japan other than the Ryukyu Islands. *Tilia maximowicziana* Shiras. (オオバボダイジュ or アオシナ) can be found in Hokkaido and northern Honshu (Tohoku region), while *T. kiusiana* (ヘラノキ) Makino & Shiras. in southern Honshu (Chugoku region) and Kyushu (Figure 1; Kurata & Hamaya, 1968–1976). In addition to the three endemic species, *T. mandshurica* Rupr. & Maxim. (including its morphologically distinct varieties: *T. mandshurica* var. *rufovillosa* (Hatus.) Kitam.—ツクシボダイジュ and *T. mandshurica* var. *inouei* Hatus.—ブンゴボダイジュ) can also be found in southern Japan; it is unclear whether *T. mandshurica* is a native or introduced species in Japan (Pigott, 2012). *Tilia miqueliana* Maxim. (ボダイジュ) was brought to Japan over 1000 years ago by monks from China and Korea during the establishment of Buddhism in the island country (Macomber, 2022; Pigott, 2012). In recent times, other *Tilia* species (e.g., *T. cordata* Mill. or *T*. × *europaea* L.) are also planted in Japanese urban environments, parks, and roads (Tanda & Nishiuchi, 2000; Tokyo Chuo City Tourism Association, 2020).

Despite the recognized economic and cultural (Forestry Agency Japan, 2012; Miyake, 2004) and suggested ecological (Janowski & Nara, 2021) importance of *Tilia* trees in Japan, they are rarely studied, and only limited information about them is available (Table 1). The primary purpose of this review is to outline the value Japanese *Tilia* trees have to people and to present this information in an organized way. Following the categorization of values trees provide to people suggested by Rivers et al. (2022), the importance of *Tilia* trees in Japan was divided into three categories: cultural and aesthetic, economic and livelihood, and ecological. Raising the awareness of trees' relevance may increase the scientific attention they receive. Moreover, summarizing this information in one place could help highlight the gaps in our current knowledge of Japanese *Tilia* trees and the *Tilia* genus in general.

**TABLE 1:** Number of scientific publications and Internet search results referencing the *Tilia* genus or selected *Tilia* species.

	General		N. America	Europe				Asia/Japan				
	*Tilia*	*Tilia Japan*	*Tilia americana*	*Tilia cordata*	*Tilia platyphyllos*	*Tilia tomentosa*	*Tilia europaea*	*Tilia japonica*	*Tilia maximowicziana*	*Tilia kiusiana*	*Tilia mandshurica*	*Tilia miqueliana*
Scopus (title, abstract, keywords)	3106	35	250	1047	257	175	107	102	6	4	147	16
Scopus (article title)	615	2	43	198	49	38	19	7	1	2	7	11
Google Scholar^a^	154,000	22,700	24,600	35,800	14,400	14,900	17,400	17,400	913	195	5480	369
Google Search^a^	16,000,000	2,270,000	1,050,000	2,370,000	868,000	838,000	769,000	525,000	14,100	5980	77,200	21,800

	Other tree genera									
	*Acer*	*Betula*	*Fagus*	*Fraxinus*	*Populus*	*Quercus*	*Abies*	*Picea*	*Pinus*	*Pseudotsuga*
Scopus (title, abstract, keywords)	12,697	14,311	14,207	8428	23,489	31,997	26,271	31,500	68,603	6207
Scopus (article title)	2243	2789	2886	1410	6487	7535	6053	6050	20,314	674
Google Scholar^a^	855,000	381,000	260,000	202,000	766,000	935,000	589,000	556,000	1,460,000	112,000
Google Search^a^	673,000,000	15,800,000	9,270,000	7,610,000	29,800,000	33,900,000	19,100,000	14,500,000	65,200,000	2,810,000

*Note*: The number of publications and Internet search results referencing other temperate climate major tree genera were provided for comparison. The results are based on searches conducted on February 18, 2023.

^a^ Approximate no. of results.

## 2 CULTURAL AND AESTHETIC IMPORTANCE

### 2.1 Religious and symbolic significance

**FIGURE 2:**
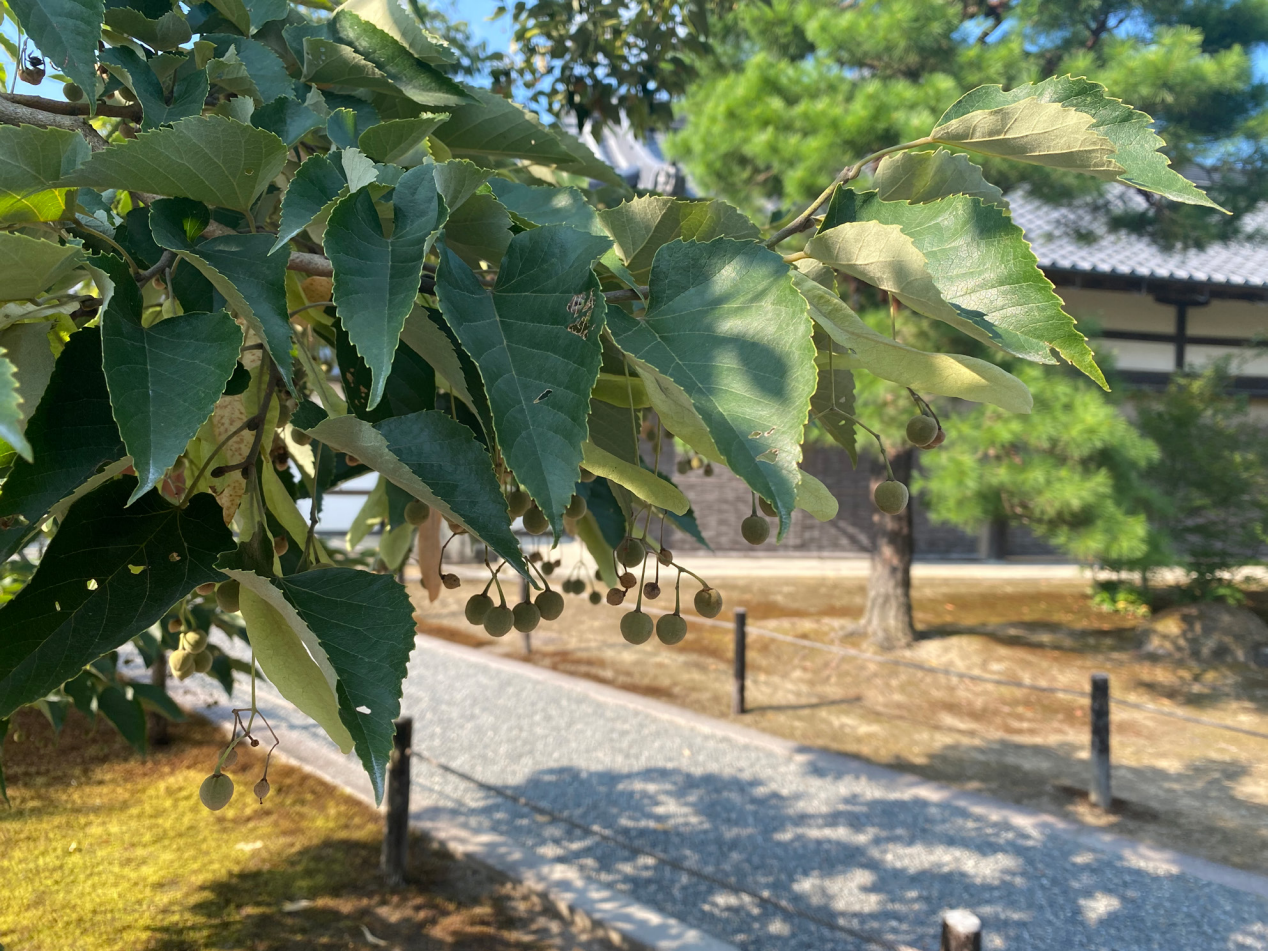
*Tilia miqueliana* (bodaiju; 菩提樹) growing in the garden of Kinkaku‐ji, one of the most famous Buddhist temples in Japan.

*Tilia* trees are important in the Japanese Buddhist tradition. According to the Buddhist teachings, the Buddha, Siddhartha Gautama, attained enlightenment by meditating under a *Ficus religiosa* tree (Gethin, 1998), referred to as pútíshù (菩提树) in Chinese and bodaiju (菩提樹) in Japanese. *Ficus religiosa*, native to the Indian subcontinent, was characteristically depicted in early Buddhist iconography as having heart‐shaped leaves. As the religion spread throughout regions of China where *F. religiosa* did not grow naturally, it was replaced with other tree species similar to the iconographic depictions, for example, *Ginkgo biloba*, *Syringa reticulata*, or *T. miqueliana* (Wang et al., 2020). After Buddhism was brought to Japan, this last tree species was also imported and planted on temple grounds as the holy bodaiju tree during the 12th century (Macomber, 2022; Pigott, 2012). Today still, *T. miqueliana* can be found growing in many Buddhist temples in Japan (Figure 2), even after *F. religiosa* trees were also brought to Japan in the 19th century (Kimura, 1958). Interestingly, while *T. miqueliana* was also often used for Buddhist sculptures in China and Korea (Choi et al., 2022; Tazuru et al., 2022), it was rarely used for this purpose in Japan. One of the few Japanese Buddhist sculptures known to be made of *Tilia* wood is the Amida‐nyorai in the Tenpourinji temple in Kobe (Kohara, 1964).

Other *Tilia* species were also sometimes used in the Buddhist context in Japan. *Tilia mandshurica* was often planted as bodaiju on temple grounds in southern Japan, being brought from the nearby Korean peninsula (Pigott, 2012). Similarly, native *T. japonica* and *T. maximowicziana* were planted in temples in northern Japan (Miya, 2016). The leaves of Japanese‐native *T. japonica* with religious inscriptions written on them were found inside a Buddha sculpture in Bujoji temple in Kyoto (Seya, 2000). Several *Tilia* trees recognized as important by local Buddhist communities can be found across Japan. These include rare *Tilia* species: *T. kiusiana* (e.g., Abu, Yamaguchi; Miyake, 2004) or *T. mandshurica* var. *inouei* (e.g., Kokonoe, Oita; Oita Prefectural Government, 1996).

Some *Tilia* trees in Japan are considered shinboku (神木), holy trees in Shinto. Shinto is a native Japanese religion that emphasizes nature worship (Bowker, 2002). According to its beliefs, some objects or places may become yorishiro (依代)—representatives, in which spirits can enter to interact with people (Okada, n.d.). Shinboku are yorishiro trees, commonly of impressive age and size. The most well‐known *Tilia* shinboku is an over 800 years old *T. japonica* in Kumanokotai Shrine in the Nagano prefecture (Kumano‐Kotai‐Jinja, n.d.; Town of Karuizawa, 2016). While *T. japonica* is the most common *Tilia* species to be recognized as shinboku (Miya, 2016), some large *T. maximowicziana* trees are also found growing at the center of Shinto shrines in northern Japan (Zenkoku Kyoju Tanbouki, 2022).

*Tilia* in Japan are also recognized for their historical and symbolic significance. *Tilia miqueliana* growing at Hoshoin temple in Hiroshima is one of the trees that have survived the explosion of an atomic bomb dropped on the city on August 6, 1945 (The City of Hiroshima Peace Policy Cross‐Sectional Committee, 2009). The city of Nagano designated *T. japonica* as its symbol (Nagano City, 2023) in relation to the historical province Shinanokuni (信濃国; currently Nagano prefecture). The province was known under this name (then written as 科野國) already in the 8th century when it appeared in Kojiki, the earliest known Japanese literary record (Suzuki, 1935). According to Ichikawa (1994), Shinanokuni (as well as many villages therein using the character 科 in their names) was named after *T. japonica* (shinanoki; 科の木). Common in the region, the tree was economically important at the time; its bast was used to weave fabrics and ropes.

### 2.2 Recreational spaces

Despite their cultural significance, *Tilia* trees are less common in cities and parks in Japan than in other parts of the world. A major exception to that is the city of Nagano, where *T. japonica*, a designated city symbol, is often seen in parks and on roadsides (Nagano City, 2023). In other Japanese cities, *Tilia* trees are usually found in lower numbers: as admixture species in parks and sporadically on roadsides. The most common urban *Tilia* species are the native *T. japonica* (Hiraoka Park, 2022; Sapporo Odori Park, 2023; Soseigawa Park, 2021; Tokyo Chuo City Tourism Association, 2020), followed by the Chinese species *T. miqueliana* (Higo‐Hosokawa Garden, 2016; Kato, 2018; The National Gardens Association, 2015; Tokyo Metropolitan Park Association, 2015) often also planted on temple grounds. The native *T. maximowicziana* can be sporadically seen in parks, especially on the northern island of Hokkaido (Sapporo Odori Park, 2022; Yurigahara Park, 2012). At the end of the 19th century, European *Tilia* species, *T. cordata*, *T. platyphyllos*, and *T*. × *europaea*, were first brought to Japan as ornamental trees (Higo, 1961). They are often planted in places attempting a Western‐like mood (Tanda & Nishiuchi, 2000; Tokyo Chuo City Tourism Association, 2020; Funabashi H.C. Anderson Park, personal communication).

## 3 ECONOMIC AND LIVELIHOOD IMPORTANCE

### 3.1 Honey production

*Tilia* trees are important honey plants in Hokkaido and northern Honshu, the primary bee‐keeping regions in Japan (Masaka, 2016; Masaka et al., 2013; Okada, 1981; Taniguchi et al., 2012). In 2021, over 530 tons of honey, 19.3% of the entire Japanese honey production, was produced in Hokkaido (Hokkaido Government Department of Agriculture, 2022). In terms of mean annual honey production, *Tilia* spp. (including *T. japonica* and *T. maximowicziana*) are the second honey plant in Hokkaido, following *Robinia pseudoacacia* (Masaka et al., 2013), a North American species introduced to Japan in the 19th century (Higo, 1961). *Tilia* spp. and *R. pseudoacacia* each contribute roughly one‐third of Hokkaido honey production (Masaka et al., 2013). However, the efficiency of honey production from *Tilia* spp. exceeds that of other honey plants in Japan, with monofloral *Tilia* honey achieving higher yields per hive per year (Masaka & Sato, 2011; Okada, 1981). The number of *Tilia* trees in an area was shown to correlate exponentially to the proportion of beehives producing monofloral *Tilia* honey in that area (Masaka et al., 2013). This suggests that if *Tilia* flowers are available, honeybees in Japan prefer them over other honey plant species. The most significant disadvantage of *Tilia* spp. to Japanese beekeepers, and one of the reasons for introducing *R. pseudoacacia*, is the unpredictability in nectar output year to year that characterizes *Tilia*. Nearly every other year, *Tilia* trees produce less than half the regular amount of nectar, and their flowers are also comparatively more vulnerable to weather (Masaka, 2016; Okada, 1981).

Similar to western *Tilia* spp. monofloral honeys (Juan‐Borrás et al., 2014), Japanese *Tilia* honey is characterized by a strong herbal smell and bright yellow color (Okada, 1981). Flavor‐wise, it is sweeter and less sour than most other Japanese honeys (Mayama et al., 1982). While the information on the composition and health benefits of Japanese *Tilia* honey is limited, more is known about *Tilia* honeys in general. They have a high mineral content (Bodó et al., 2021; Juan‐Borrás et al., 2014; Oroian et al., 2015). Although the antioxidant properties of *Tilia* honeys are average compared with other monofloral honeys, they much exceed the more commercially popular *R. pseudoacacia* honey (Bodó et al., 2021). *Tilia* honeys have one of the strongest antibacterial properties (Balázs et al., 2021). Due to their calming properties, *Tilia* honeys are also used to alleviate insomnia and stress (Yaniv & Rudich, 1997).

### 3.2 Pharmacological products

Plants in the *Tilia* genus are known for their high content of bioactive secondary metabolites. While most studies of pharmaceuticals in *Tilia* were done on European taxa, several unique compounds were found in *T. japonica* and *T. kiusiana*. Tilianin (acacetin 7‐*O*‐glucoside), a potential drug preventing cardiovascular disorders (Khattulanuar et al., 2022), was first discovered in the leaves of *T. japonica* (Nakaoki et al., 1960). Another active compound Nakaoki et al. (1960) found in *T. japonica* leaves was rutin (quercetin 3‐*O*‐rutinoside), shown to have cytoprotective and anti‐inflammatory properties (Hosseinzadeh & Nassiri‐Asl, 2014). Found in and named after *T. kiusiana*, kiusianins are sterol compounds cytotoxic to human cancer cells (Shimada et al., 2014). More flavonoids with potential medicinal effects were reported from other *Tilia* species (Iwashina & Kokubugata, 2012).

While *Tilia* herbal materials are used in Chinese and European traditional medicine (Liu et al., 2012; Poetzsch, 2016; Yaniv & Rudich, 1997), it is less prominent in Japanese traditional remedies. In Japan, herbal teas made of dried *T. japonica* and *T. miqueliana* flowers are traditionally used to lessen cold symptoms (Hirobe, 2000; Watanabe, 2003). The calming effect of *Tilia* spp. leaves and flowers is also acknowledged in Japanese sources (Shoyama, 2014). Recently, extract from *Tilia* flowers is used in Japanese cosmetics and skin care products (Ichimaru Pharcos Co., Ltd., 2012; Watanabe, 2003).

### 3.3 Wood

While *Tilia* wood (basswood) has an exceptionally low density and is soft for hardwood (Brush, 1922), *T. japonica* has relatively high wood density among the *Tilia* species. With an average density of around 450 kg/m^3^ (Fukazawa & Ohtani, 1979), its wood is denser than *T. americana* (around 380 kg/m^3^; Brush, 1922) and is comparable with the European *T. cordata* (410–500 kg/m^3^; Dünisch, 2017). Japanese basswood is traditionally used for cabinets, wooden boxes, small, light items (e.g., chopsticks and pencils), or kyougi (経木)—thin layers of wood used as a substitute for paper and for packaging. It is also an important wood used for plywood and veneers (Japan Wood Products Information & Research Center, n.d.; Yahagi, 2018). Plywood and veneers made with *T. japonica* wood are of higher quality and better adhere compared with those made with Chinese *Tilia* species (Kishino & Nakano, 2003). Due to its softness, in Hokkaido *T. japonica* wood is often used to carve commemorative bear sculptures (Shibuya et al., 2019).

### 3.4 Bast

*Tilia* spp. used to be an important fiber crop in Japan, and its bast fibers used by both the Japanese people and the Ainu people of Hokkaido. Peeling the bark of *Tilia* spp. is easier and can be done for longer periods each year (until mid‐July) compared with other tree species (e.g., *Ulmus laciniata* and *Ulmus davidiana* var. *japonica*) that were used for bast in Japan (Sato, 2018). The Ainu people utilized bast of *T. japonica* and *T. maximowicziana*, the latter being less popular for its lower quality (Saito, 1995). They used *Tilia* bast for various purposes, for example manufacturing clothes, carrying bags, floor mats, ropes, and fishing nets (Hitchcock, 1892; Saito, 1995). Attus, the Ainu fabric used for clothing resembling Japanese kimono, was typically weaved either from *U. laciniata* or *T. japonica* bast (Sato, 2018).

The ethnic Japanese people used to weave *T. japonica* bast cloth called shinafu (シナ布), a smooth, elegant fabric resistant to wrinkling (Tamaki, 1971; Yamanaka et al., 2003). Manufactured in different forms since the Jomon period (14000–300 BCE), shinafu is one of the oldest types of fabric continually produced and used in Japan (Yamanaka et al., 2003). Shinafu was not the only *Tilia* material used by the Japanese people to make clothing. Due to their waterproof properties, wide straps of bast were bound together to make raincapes used by farmers (Pigott, 2012). Other than for clothes, the Japanese used *T. japonica* bast fabric to produce straining cloths traditionally used in alcohol production. In northern Honshu, *T. maximowicziana* bast cloth was used for traditional bags for soy sauce fermentation (Yanagita, 1941). On the island of Kyushu, *T. kiusiana* and *T. mandshurica* var. *rufovillosa* were primarily used instead of *T. japonica*. *Tilia mandshurica* var. *rufovillosa* was particularly preferred by the local people who often planted it close to their rice fields to easily collect the bark (Pigott, 2012; Yanagita, 1941).

In response to the post‐war economic growth, many people in Japan pursued office jobs at the cost of traditional occupations. This has led to the number of people producing *Tilia* bast fabrics drastically decreasing, resulting in a decrease in the fabric's availability and an increase in its price (Tamaki, 1971). Currently, both attus and shinafu are included on the list of Japan's Nationally Designated Traditional Craft Products (Ministry of Economy Trade and Industry, 2022), recognized and protected by the Dentōteki Kōgeihin Sangyō no Shinkō ni Kansuru Hōritsu (Traditional Industries Law; 伝統的工芸品産業の振興に関する法律). Together with the recently growing appreciation of traditional Japanese crafts, the production of bast fabrics in Japan is being revitalized, and a growing number of manufacturers specializing in shinafu are opening and can be found online.

## 4 ECOLOGICAL IMPORTANCE

### 4.1 Pollinators

*Tilia* spp. are generalists in terms of pollination. *Tilia* flowers are shallow, allowing most insect pollinators to access the nectar. They produce large volumes of nectar with high sugar content. The flowers are open and produce nectar both during the day and night (Anderson, 1976; Pigott, 2012). With few differences in flower morphology between species, pollinators do not differentiate between *Tilia* species (Anderson, 1976). While bees (mainly *Andrena* spp., *Apis* spp., and *Bombus* spp.; also *Lasioglossum* spp.) are the largest and most conspicuous group of insects visiting *Tilia* flowers, hoverflies (Syrphidae) are another significant group of diurnal pollinators, while moths (e.g., Crambidae and Noctuidae) are important nocturnal pollinators (Pigott, 2012).

Although some studies of pollination in Japanese *Tilia* spp. are available, they focus on flower physiology and provide only limited information on the visiting pollinators (Ito & Kikuzawa, 1999, 2000, 2003). The pollination ecology of Japanese *Tilia* spp., especially in southern Japan, is largely underexplored. Insects reported to visit *Tilia* flowers in Japan are summarized in Table 2.

**TABLE 2:** Insects recorded visiting flowers of Japanese *Tilia* spp.

Order	Genus	Species
Hymenoptera	*Andrena* (1)	
	*Apis* (1)	*A. cerana japonica* (2)
		*A. mellifera* (2, 3, 6)
	*Bombus* (1, 6)	*B. ardens* (3, 4, 5)
		*B. diversus* (4, 5)
		*B. hypocrita* (3, 5)
	*Lasioglossum* (1)	
	*Vespa* (6)	
	*Xylocopa*	*X. appendiculata circumvolans* (3)
Lepidoptera	*Graphium*	*G. sarpedon* (7)
	*Papillo*	*P. protenor* (7)
		*P. xuthus* (7)
	*Rapala*	*R. arata* (7)
	*Vanessa*	*V. indica* (7)
	Unidentified Papilionoidea (6)	
Diptera	Unidentified Brachycera (6)	
	Unidentified Nematocera (6)	
	Unidentified Syrphinae (1)	
Hemiptera	Unidentified Pentatomoidea (6)	
Coleoptera	Unidentified Coleoptera (6)	

*Note*: Numbers in parentheses indicate sources. Source numbers next to genus names indicate that the source did not identify the insects to the species level.

*Source*: 1—Ishida and Nagasaka (1996); 2—Masaka et al. (2013); 3—Ito and Kikuzawa (2003); 4—Mizui (1993); 5—Inari et al. (2012); 6—Ito and Kikuzawa (1999); 7—Matsuda (2005).

Studies of *Bombus* spp. in northern Japan identified *T. japonica* and *T. maximowicziana* as crucial sources of nectar for these ecologically and economically essential pollinators (Inari et al., 2012). *Tilia* flowers provide *Bombus* spp. a source of food in summer after many other nectar flowers have finished blossoming. *Tilia* nectar is especially vital to *Bombus diversus*. This species develops colonies later than other Japanese *Bombus* spp., and the availability of *Tilia* flowers is an important determinant in the number of colonies established.

### 4.2 Ectomycorrhizal fungi

*Tilia* is the only known ectomycorrhizal genus in the family Malvaceae (Smith & Read, 2008). *Tilia* spp. host a wide range of ectomycorrhizal fungal species, both in natural forests (Janowski & Nara, 2021; Lang et al., 2011; Timonen & Kauppinen, 2008) and urban spaces (Csizmár et al., 2021; Timonen & Kauppinen, 2008; Van Geel et al., 2018). Planting *Tilia* trees in cities may potentially lead to an increased diversity of soil fungi (Csizmár et al., 2021; Janowski & Leski, 2022), indirectly improving the soil condition. Several studies indicated that *Tilia* spp. readily associate with the *Tuber* genus, with some suggesting that *Tilia* trees could be considered for use in truffle orchards (Rudawska et al., 2019; Timonen & Kauppinen, 2008).

As the majority of *Tilia* ectomycorrhizal relation studies are conducted in Europe, the ectomycorrhizal relations of Japanese *Tilia* spp. are not well known. A single study of ectomycorrhizal communities hosted by *T. japonica* in Japanese forests revealed a high diversity of fungal partners, many of which were previously unreported or species first recorded in Japan (Janowski & Nara, 2021). Janowski and Nara (2021) suggest that *T. japonica* might be playing a significant role in the local ectomycorrhizal networks, increasing their connectance and stability.

## 5 SUMMARY

Although Japanese *Tilia* spp. are undoubtedly important trees, they are understudied on many fronts. Compared with European *Tilia* spp., few studies evaluate the pharmacological applications of Japanese *Tilia* spp. herbal material. This is despite the few available studies confirming *T. japonica* and *T. kiusiana* to contain multiple compounds with potential medical uses. Limited information is available on the properties of Japanese *Tilia* honey, and no studies comparing it to European *Tilia* honey are available. However, practical information on the use of *Tilia* in Japanese apiculture is relatively comprehensive. Close to no information is available about Japanese *Tilia* herbal teas, despite their traditional consumption in Japan. *Tilia* bast fiber collection and preparation, together with the history and properties of *Tilia* bast fabrics, are the best‐represented application of Japanese *Tilia* spp. in literature.

The ecology of Japanese *Tilia* spp. is particularly underexplored. No studies concerning the ecology of the southern Japanese taxa, *T. kiusiana*, *T. mandshurica* var. *rufovillosa*, and *T. mandshurica* var. *inouei*, are available to the author's knowledge. Most available data on their distribution is old and often outdated (personal communication). In many cases, *Tilia* trees have been removed and replaced with timber plantations or roads. Even the relatively better‐known *T. japonica* and *T. maximowicziana* receive only limited attention. Currently, *T. mandshurica* var. *rufovillosa* is recognized as an endangered species on the national level, while all other native Japanese *Tilia* species are recognized as endangered or critically endangered in individual prefectures (Association of Wildlife Research, 2021; Ministry of the Environment, 2020). Renewed scientific attention would be required to better evaluate the status of *Tilia* spp. in Japan and ensure the genus' conservation.

## AUTHOR CONTRIBUTIONS

**Daniel Janowski:** Conceptualization (lead); formal analysis (lead); investigation (lead); writing – original draft (lead); writing – review and editing (lead).

## Data Availability

No new data was created in this study.
